# Nutrition deficiency increases the risk of stomach cancer mortality

**DOI:** 10.1186/1471-2407-12-315

**Published:** 2012-07-28

**Authors:** Qing Da Li, Hao Li, Fu Ji Li, Mei Shu Wang, Zhuo Jian Li, Jing Han, Qing Hui Li, Xiang Ji Ma, Da Nan Wang

**Affiliations:** 1 Department of Medicine, Qilu Hospital of Shandong University, Jinan, China; 2 Tumor Center, Qilu Hospital of Shandong University, Jinan, China; 3 Department of Epidemiology, College of Public Health of Shandong University, Jinan, China; 4 Center of Disease Control and Prevention of Jinan, Jinan, China; 5 Department of Environmental Medicine, School of Medicine New York University, New York, USA; 6 Department of Epidemiology, Institute of Basic Medicine of Shandong Academy of Medical Sciences, Jinan, China; 7 Center of Disease Control and Prevention of Shandong Province, Jinan, China; 8 Center of Disease Control and Prevention of Zhaoyuan, Zhaoyuan, China

**Keywords:** Nutritive deficiency, Stomach Cancer, Mortality, Famine exposure

## Abstract

**Background:**

The purpose of the study is to determine whether exposure to malnutrition during early life is associated with increased risk of stomach cancer in later life.

**Methods:**

The design protocol included analyzing the trend of gastric cancer mortality and nutrition and evaluating the association between nutrient deficiency in early life and the risk of gastric cancer by hierarchical age–period–birth cohort (APC) analysis using general log-linear Poisson models and to compare the difference between birth cohorts who were exposed to the 1959–1961 Chinese famine and those who were not exposed to the famine. Data on stomach cancer mortality from 1970 to 2009 and the dietary patterns from 1955 to 1985 which included the 1959–1961 Chinese famine period in the Zhaoyuan County population were obtained. The nutrition information was collected 15 years prior to the mortality data as based on the latest reference of disease incubation.

**Results:**

APC analysis revealed that severe nutrition deficiency during early life may increase the risk of stomach cancer. Compared with the 1960–1964 birth cohort, the risk for stomach cancer in all birth cohorts from 1900 to 1959 significantly increased; compared with the 1970–1974 cohort, the risk for stomach cancer in the 1975–1979 cohort significantly increased, whereas the others had a steadily decreased risk; compared with 85–89 age group in the 2005–2009 death survey, the ORs decreased with younger age and reached significant levels for the 50–54 age group after adjusting the confounding factors. The 1930 to 1964 group (exposed to famine) had a higher mortality rate than the 1965 to 1999 group (not exposed to famine). For males, the relative risk (RR) was 2.39 and the 95% confidence interval (CI) was 1.51 to 3.77. For females, RR was 1.64 and 95% CI was 1.02 to 2.62.

**Conclusion:**

The results of the present study suggested that prolonged malnutrition during early life may increase the risk of stomach cancer mortality in later life.

## Background

The incidences and mortality rates of gastric cancer have decreased worldwide over the past decades [1-4]. Nevertheless, this cancer remains the second most important cause of cancer death, especially in China [5,6]. The relationship between nutritional conditions of early life and mortality due to gastric cancer in later life has been the subject of debate. Some studies have observed the association between gastric cancer and substandard living conditions, including a high infant mortality around the time of birth [7,8] as well as low socio-economic gradients and circumstances during early life [9-12]. In contrast, no association was observed in other studies [13]. During infancy and childhood, factors such as salt consumption, vitamin C intake [14], and *Helicobacter pylori* (*H. pylori*)infection [15] may be linked to the etiology of stomach cancer.

A recent study has revealed that several citrus components strongly suppress CD74 (a new receptor for *H. pylori* urease) expression in the gastric carcinoma cell line. Auraptene (citrus coumarin) is found to disrupt serum starvation-induced extracellular signaling-regulated kinase 1/2 activation, and attenuate *H. pylori* adhesion as well as IL-8 production in a co-culture system [16]. Favorable developments in childhood nutrition may have contributed to the obvious decline in stomach cancer mortality.

There is some evidence in population health literature showing that exposure to malnutrition in the fetal period and early childhood exerts significant lasting effects on health [17,18]. Famines provide a quasi-experimental setting for observation of the long-term effects of nutritional deprivation on human development. Exposure to famine in early life is associated with an increased risk of metabolic syndrome development in later life, which included hypertension [19-21], insulin resistance [22-25], central obesity [26-28] and dyslipidemia [29,30]. One current study provided further evidence that both fetal and infant exposure to severe famine increased the clustering of the metabolic risk factors that predispose a person to type 2 diabetes and cardiovascular disease [31]. To date, linkage of exposure to the Chinese famine with the risk of stomach cancer in later life has not been reported.

The 1959–1961 Chinese famine was the largest in human history, and affected all of China. The genesis of the famine was the “Great Leap Forward” campaign launched by Mao in 1958 [32,33]. Zhaoyuan County, one of the most severely affected rural counties at that time, was among the places in China with the highest stomach cancer mortality rates according to the first cancer death survey conducted from 1970–1974 in Shandong Province. Since then, data on all causes of deaths in the area have been monitored [34].

The aim was to describe the mortality trends among successive birth cohorts considering malnutrition in the early decades of their lives. The main hypothesis was that malnutrition during childhood or early life is associated with increased risk for developing stomach cancer in later life. We used data on the stomach cancer mortality prevalence in a population at risk for high mortality, i.e., the Zhaoyuan County population. Using these data, we evaluated the stomach cancer mortality trends in the different cohorts. Exposure to the Chinese famine was found to correspond closely with long-term malnutrition during early life.

## Methods

### Study design

The human subject protocol for this study was approved by the Ethics Committee of the Medical Faculty of the Shandong Academy of Medical Sciences.

The design of the present study consisted of three parts: (1) to analyse the trend of gastric cancer mortality during 1970–2009 in Zhaoyuan County and to estimate the least reference of the incubation period of gastric cancer; (2) to evaluate the efficacy of nutrient deficiency and whether it is linked with the risk of gastric cancer by hierarchical age-period-cohort (APC) analysis of the available surveillance data; (3) to compare the difference in the risk of gastric cancer mortality between the birth cohorts who were exposed to the 1959–1961 Chinese famine and the birth cohorts who were not exposed to the famine.

### Data of mortality stomach cancer

Data on the number of deaths due to stomach cancer in the population between 1970 and 2009 were obtained. Data for the period of 1970 to 1984 came from three retrospective death surveys (1970–1974, 1975–1979, and 1980–1984) carried out by our research group [6]. Data for the period of 1985 to 2009 came from the death registration division of the Center of Disease Control and Prevention of Zhaoyuan County, which only started registering deaths in 1985.

In order to control the data quality, an internal procedural check system, which evaluated timeliness of death registration, completeness of entries in the registration form, and the accuracy of data entry errors were corrected through re-checking the hospital records for each death. Data validity and completeness were evaluated using indices such as the proportion of microscopically verified cases (MV%), the proportion of death-certificate-only registrations (DCO%), and the mortality to incidence ratio (M: I) [35]. For the eight death survey periods (1970–1974, 1975–1979, 1980–1984, 1985–1989, 1990–1994, 1995–1999, 2000–2004, and 2005–2009), the MV% values were 42.7%, 43.5%, 45.7%, 44.3%, 47.5%, 49.8%, 47.3%, and 48.2%, respectively. The DCO% values were 2.1%, 1.8%, 2.5%, 1.9%, 1.0%, 0.9%, 0.8%, and 0.02% respectively. Other cases were diagnosed by gastroscopy or X-ray barium meal examination without surgical treatment. The M: I values were between 70.0 and 75.0 from 1985 to 2009. This is consistent with the report from Qidong County of China [36]. Rao et al. (2007) used the hospital codes for each death as a standard classification system to assess validity of registration diagnoses for urban China and the validation sensitivity of stomach cancer was 91.7% (95% confident interval:86-97%) [37]. The validation sensitivity of gastric cancer in Zhaoyuan County was 90.1% - 95.6% during the study period.

The code corresponding to stomach cancer in the International Classification of Diseases was 151 in the 9th revision (1979–1994) and C16 in the 10th revision (1995–2009). Based on these data, the age-specific mortality by gender was calculated for 13 five-year age groups (20 to 84) and 8 five-year periods (1970 to 2009). Using these classifications, 20 overlapping five-year birth cohorts were identified and defined according to the central year of the birth cohort. Crude and age-specific rates were calculated for 8 five-year time periods between 1970 and 2009. The age-standardized rates were calculated by the direct method using the world standard population (National Office for Cancer Prevention and Control, 1980).

### Data of malnutrition

Based on the present study data of gastric cancer, we estimated that the least reference of incubation period of gastric cancer was 15 years (shown below). The nutrition information was collected 15 years prior to the gastric cancer mortality data.

Data on the grain per capita from 1955 to 1985 for farmers came from the historical records of Zhaoyuan County Annals [38]. The nutritional components including calocalorie, protein, fat, and carbohydrate were calculated using a method from the book entitled “China Food Composition 2004” [39].

The malnutrition criterion was based on the study of Pertha and Debraj (1986) [40], who stated that “the average population caloric need for productive agricultural laborers (or for normal child development) is 1870.7 calories per day, and the average need to stay alive is approximately 804.4 calories per day.”

Regarding the food consumption pattern of the studied population, vegetables were the priority. About 90%caloric and more than 80% protein intakes were from vegetables before the economy was reformed and opened [41,42]. Therefore, the total average grain per capita was used to estimate the nutritional components in the present study. There was no available historical data on animal meat in the annals of the county. Hence, we adjusted the caloric and protein consumptions by a multiplier of 1.10 (i.e., equal to 1 divided by 90% or 80%).

### Famine severity

First, we adopted the approach used by Chen and Zhou (2007), who used excess mortality in 1959–1961 at the province level to generate a measure of severity of the famine [20]. The excess mortality was calculated as the difference between death rates in the famine year and the average of death rates in 1956–1958. Table 1 shows the mortality rates of 1955 to 1966 and the excess death rates of 1959–1963 in Zhaoyuan County. The death rates were relatively stable prior to 1959, but during 1959–1963, death rates rose sharply and were on average significantly higher than the years before and after the famine. The time period (1959–1963) of the famine in Zhaoyuan County was longer than the average 3 years (1959–1961) of the famine in the whole of Shandong Province. The worse period of the famine in Zhaoyuan County was also from 1959 to 1961.

**Table 1:** Death rates of 1955–1966 and excess death rate of 1959–1962 and the number of person born during 1956–1964 in Zhaoyuan

	**1956**	**1957**	**1958**	**1959**	**1960**	**1961**	**1962**	**1963**	**1964**	**1965**	**1966**
**Panel A: Death rates in Shandong Province and Zhaoyuan County**											
Sandong ^a, b^	12.1	12.1	12.8	18.2	23.6	18.4	12.4	11.8	12.0	10.2	9.9
Zhaoyuan	13.7	13.6	13.5	19.0	21.4	21.7	18.1	16.6	14.2	12.3	10.8
**Panel B:**^**a**^**Excess death rates in Shandong Province and Zhaoyuan County**											
Sandong Province				5.9	11.3	6.07					
Zhaoyuan				5.4	7.8	8.1	4.5	3.0	0.6		
**Panel C: The number of person born during 1956–1964 in Zhaoyuan County**^**b**^											
Cohort (born year)	1956	1957	1958	1959	1960	1961	1962	1963	1964		
population	12638	14732	10856	10751	11364	9393	16899	20176	17602		
CSSI^c^	0.66										

^a^: The excess death rates in Shandong Province is adapted from Chen and Zhou (2007), Table 1, P664.

^b:^ Data source: Shandong Bureau Statistics and Population Census Office. 1949–1984 A Compilation of Population Statistical Data. Jinan, Shandong Bureau Statistics Jining Press 1985.

^c:^ Abbreviation used: CSSI, cohort size shrinkage index.

Second, we used the method put forward by Huang, et al. (2010), who used cohort size shrinkage index (CSSI) to evaluate the famine severity for each county in China [21]. The CSSI = (N_nonfamine_ – N_famine_)/N_nonfamine_. Where the N_famine_ is the mean cohort size of a person born during the famine years (1959–1961) and the N_nonfamine_ is the mean cohort size of a person born during the 3 years immediately before the famine (1956–1958) and the 3 years immediately after the famine (1962–1964) in Zhaoyuan County [43]. As shown in Table 1, the CSSI was 0.66 for the famine in Zhaoyuan County, and it was near the highest value of 35 counties famine indexes (from 0.24 to 0.64) reported by Huang, et al. [21].

### Statistics

The annual percent change was calculated using the Joinpoint regression model. The software used enabled the determination of the statistical significance of an apparent change in trend [44]. The frequencies of the incubation periods for gastric cancer were groups of 5-year intervals, the cumulative percentages were determined at these intervals and the logarithms of these values were plotted on normal probability graph paper. The value of the median was the incubation period of gastric cancer to be estimated following exposure to the 1959–1963 Chinese famine in Zhaoyuan County [45].

To evaluate the independent variables (age, period, birth cohort, and famine) associated with the risk of stomach cancer, general log-linear Poisson models were fitted by a maximum likelihood method. The stomach cancer mortality was assumed to follow a Poisson distribution. Each factor in the models had an additive effect on the log rate as follows:

(1) $$\log\lambda_{\mathrm{ijk}}=\mu +\alpha_{i}+\pi_{j}+\gamma_{k}+x+z_{j}+\epsilon_{\mathrm{ijk}}\text{,}$$

Where the age effect is represented by *α*_*i*_ (*i* = 1,…, 13), the period effect is *π*_*j*_ (*j* = 1,…, 8), the birth cohort effect is *γ*_*k*_ (*k* = 1,…, 20), the gender effect is *x*, and the famine effect is *z*_*j*_ (*j* = 0, 1). *ϵ*_*ijk*_ represents the random error.

In the general log-linear Poisson model, the dependent variable was the number of deaths, with the person-years at risk as the offset variable. As independent variables, we included age and birth cohorts with five-year intervals and eight periods of five-year intervals from 1970 to 2009. The parameters were estimated as the relative risk (OR) using data on the 80–84 age group, 1970–1974 death survey period, and 1890–1894 birth cohort as the reference values (1.0).

To evaluate the independent variables, i.e., age–period–birth cohort (APC), famine, and gender associated with the risk of stomach cancer, three general log-linear Poisson models were fitted by a maximum likelihood method. In these models, the dependent variable was the number of stomach cancer deaths, with the person-years at risk as the offset variable. In one model, the independent variables included the factor of period as well as covariates of gender, age, and famine. The second model included the factor of age as well as covariates of gender, period, and famine. The third model included the factor of birth cohort as well as covariates of gender, period, and famine.

The ORs for these parameters were adjusted with respect to other parameters as derived from the best-fit model. There was a high correlation between the age and birth cohort. Thus, to avoid the colinearity in the APC regression model, the two variables were not included in the same regression model.

To evaluate the long-term effect of famine on stomach cancer, we compared the stomach cancer deaths between two groups with and without exposure to malnutrition. The former included 7 five-year birth cohorts from 1930 to 1964, and was compared with the death data from 1970–1974. The latter included 7 five-year birth cohorts from 1965 to 1999, and was compared with the death data from 2005–2009. Statistical analysis was conducted using SPSS version 17.0.

## Results

### Descriptive analysis

**Figure 1:**
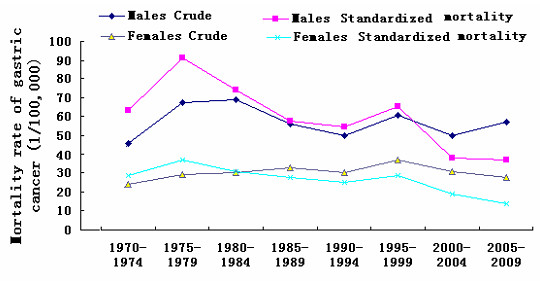
The mortality rates of males and females during 1970–2009 in Zhaoyuan County (Note: The standardized mortality was calculated by the percentages of world population in 1980).

The accumulative total population in Zhaoyuan County was 2,411,412 during the 1970–1974 death survey and 2,830,866 during the 2005–2009 death survey. The stomach cancer mortality per 100,000 individuals by gender and survey periods is shown in Table 2 and Figure 1. The respective crude and standardized mortality rates of stomach cancer were 34.94 and 44.82 per 100,000 individuals in the 1970–1974 death survey and 42.20 and 24.43 per 100,000 individuals in the 2005–2009 death survey. Despite an increase in stomach cancer mortality in the 1970–1974 and 1975–1984 death surveys, a remarkable general decrease was noticed from 1985 to 2009. Using the Jionpoint regression models, the annual percent change in stomach cancer mortality rates was −2.64% (95% confidence interval, CI = −3.88% and −1.37%) from 1985 to 2009. The annual changes were −2.07% (95% CI = −3.19% and −0.94%) for males and −2.09% (95% CI = −3.11% and −1.06%) for females during the same period. The stomach cancer mortality peaked in 1975–1979. This period was 15 years to 20 years after the 1959–1961 Chinese famine, which ended in 1963. For the present data following exposure to the 1959–1961 Chinese famine, the incubation period of gastric cancer was estimated as 17.3 (99% confident interval:15.5-19.1) years.

**Table 2:** Mortality rates of stomach cancer during the eight periods of 1970–2009 in Zhaoyuan County (Rate per 100,000)*

**Period**	**Males**		**Females**		**Total**	
	**Crude mortality**	**Standardized mortality**	**Crude mortality**	**Standardized mortality**	**Crude mortality**	**Standardized mortality**
1970-1974	45.98	63.11	24.02	28.82	34.94	44.82
1975-1979	67.27	91.47	28.83	36.76	48.13	68.24
1980-1984	68.73	74.22	30.21	30.59	49.54	51.50
1985-1989	56.29	57.9	32.44	27.45	44.34	41.55
1990-1994	50.16	54.66	29.90	25.01	40.05	42.05
1995-1999	60.55	65.31	36.83	28.42	48.73	43.63
2000-2004	49.78	37.61	30.67	18.70	40.18	27.37
2005-2009	57.02	36.48	27.50	13.91	42.20	24.43

*: Standardized mortality calculated in direct method by world standard population.

**Figure 2:**
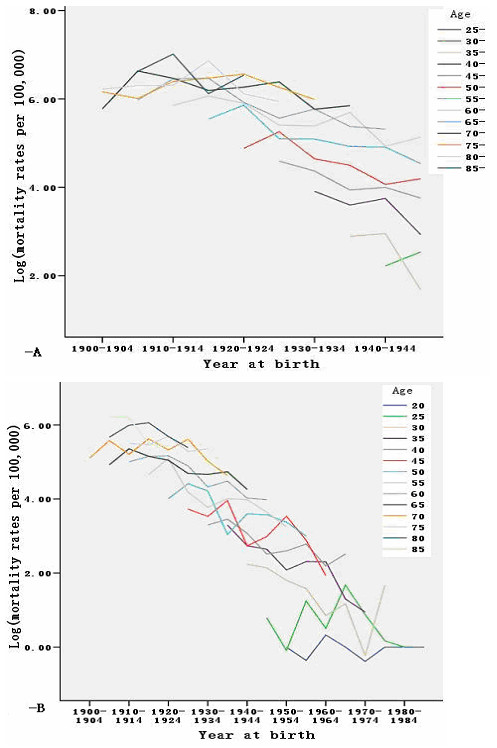
**A Age-specific mortalities of stomach cancer by the year of birth for males during the period of 1970–2009; Figure **2**-B Age-specific mortalities of stomach cancer by the year of birth for females during the period of 1970–2009.**

Figures 2-A and 2-B show the age-specific mortality rates of males and females for the different birth cohorts. The age-specific mortality rate of stomach cancer decreased in the more recent birth cohorts. Within each birth cohort, the mortality rates of stomach cancer were found to decrease with age.

**Figure 3:**
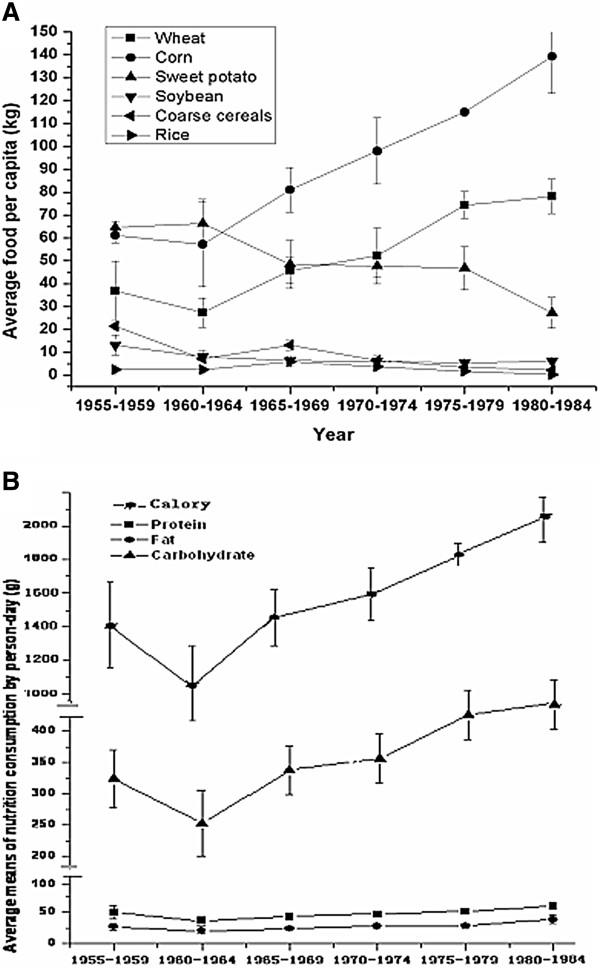
**A The average means of food consumption per capita for rural residents during 1955–1985 in Zhaoyuan County.** Figure 3-B The average means of nutrition consumption per capita for rural residents during 1955–1985 in Zhaoyuan County. Note: caloric unit is kilo-calorie.

The average farmer’s food consumption per capita from 1955 to 1985 in Zhaoyuan County is shown in Figure 3-A. Figure 3-B shows the average calorie, carbohydrate, protein, and fat per capita per year of Zhaoyuan residents from 1950 to 1985. The average caloric levels from 1960 to 1961 were near the average level of survival for the residents.

### Risk factors associated with stomach cancer mortality by a log-linear general Poisson regression model

**Figure 4:**
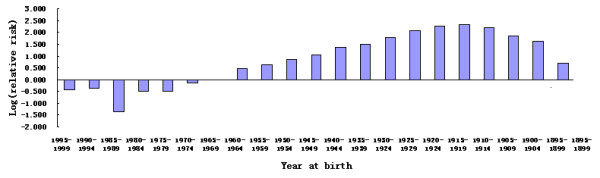
Relative risks of birth cohorts on stomach cancer mortality compared to the birth cohort of 1960–1964 as the baseline.

As shown in Table 3 and Figure 4, the results of the analysis using a log-linear general Poisson regression model indicated the following: (1) compared with the 1960–1964 birth cohort, the risk for stomach cancer in all birth cohorts from 1900 to 1959 significantly increased after adjusting gender, age, period, and famine factors; (2) compared with the 1970–1974 cohort, the risk for stomach cancer in the 1975–1979 cohort significantly increased, whereas the others had a steadily decreased risk; (3) compared with 85–89 age group in the 2005–2009 death survey, the ORs decreased with younger age and reached significant levels for the 50–54 age group (*P* < 0.005); and (4) compared with females, males had increased risk for stomach cancer (OR = 2.31; 95% CI = 2.22 and 2.41) after adjusting the confounding factors.

**Table 3:** **The relative risk of stomach cancer mortality for famine and age-cohort-period analysis**^**a**^

**Factor**	**OR(95%CI)**	**Factor**	**OR(95%CI)**	**Factor**	**OR(95%CI)**
**Age**		**Cohort**		**period**	
20-	0.02(0.00-0.11)	2005-2009	0.00	2005-2009	0.99(0.41-2.39)
25-	0.04(0.01-0.20)	2000-2004	0.00	2000-2004	0.84(0.39-1.84)
30-	0.08(0.02-0.33)	1995-1999	0.66(0.08-5.70)	1995-1999	1.11(0.56-2.20)
35-	0.11(0.03-0.41)	1990-1994	0.19(0.03-1.24)	1990-1994	0.77(0.42-1.40)
40-	0.19(0.06-0.60)	1985-1989	0.71(0.15-3.35)	1985-1989	0.68(0.40-1.16)
45-	0.28(0.10-0.78)	1980-1984	0.26(0.06-1.16)	1980-1984	0.73(0.45-1.18)
50-	0.36(0.15-0.88)	1975-1979	0.62(0.31-1.25)	1975-1979	1.42(1.22-1.66)
55-	0.51(0.24-1.10)	1970-1974	0.60(0.36-0.99)	1970-1974	1.00
60-	0.68(0.36-1.29)	1965-1969	0.88(0.63-1.23)		
65-	0.76(0.46-1.29)	1960-1964	1.00		
70-	0.96(0.65-1.43)	1955-1959	1.60(1.22-2.08)		
75-	0.93(0.70-1.23)	1950-1954	1.89(1.35-2.66)		
80-	0.97(0.81-1.17)	1945-1949	2.32(1.50-3.60)		
85-	1.00	1940-1944	2.83(1.64-4.90)		
		1935-1939	3.92(2.02-7.64)		
		1930-1924	4.54(2.06-9.97)		
		1925-1929	6.01(2.42-14.92)		
		1920-1924	8.02(2.85-22.56)		
		1915-1919	9.58(3.01-30.54)		
		1910-1914	10.15(2.81-36.60)		
		1905-1909	9.20(2.25-37.64)		
		1900-1904	6.36(1.37-29.58)		
		1895-1899	5.12(0.95-27.61)		
		1895-1899	2.02(0.32-12.73)		

^a^: adjusted for famine and gender; 95% CI = 95% Confidence Interval. ^b^ : Age 85- years at the period 2005–2009 as the baseline in the age model. ^c^ : the period 1970–1974 as the baseline in the period model; ^d^ : the birth cohort 1960–1964 as the baseline in the birth cohort model.

### Comparison of the risk for stomach cancer between the two groups with and without exposure to the Chinese famine

As shown in Table 4, the standardized mortality rates of stomach cancer in the 7 five-year birth cohorts from 1930 to 1964 (exposed to famine) were 10.26 per 100,000 for males and 2.20 per 100,000 for females in the 1970–1974 death survey. However, the standardized mortality rates of stomach cancer in the 7 five-year birth cohorts from 1965 to 1999 (not exposed to famine) were 7.67 per 100,000 for males and 2.34 per 100,000 for females in the 2005–2009 death survey period. The group exposed to famine had an increased risk for stomach cancer, with relative risk (RR) values of 2.39 (95% CI = 1.51 to 3.77) for males and 1.64 (95% CI = 1.02 to 2.62) for females.

**Table 4:** **Stomach cancer mortality compared between famine exposure for the birth cohorts of from 1960–1964 to 1930–1934 in survey time of 1970–1974 and no famine exposure the birth cohorts of from 1995–1999 to 1965–1969 in survey time of 2005-2009**^**a**^

**Survey time**	**Seven 5-year birth cohorts**	**Famine exposure**	**Population**	**all cause deaths**	**Stomach cancer deaths**	**Crude stomach cancer mortality**	**Standardized stomach cancer mortality**^**b**^
Males:							
1970-1974	1930-1934 to 1960-1964	Yes	727391	1160	63	8.66	10.26
2005-2009	1965-1969 to 1995-1999	no	716785	1352	26	3.63	2.20
Females:							
1970-1974	1930-1934 to 1960-1964	Yes	700724	889	45	6.42	7.67
2005-2009	1965-1969 to 1995-1999	no	713375	530	28	3.93	2.34

^a^: When comparing the rates of stomach cancer mortality of born in 1965–1999 who without exposure to the famine with that of born in 1930–1964 who exposure to the famine, the Pearson Chi-square: 14.815, P < 0.001 for males, and that the Pearson Chi-square: 4.269, P = 0.039 for females, respectively. ^b^: Standard mortality was calculated by the world population of National Office for Cancer Prevention and Control. 1980, and the unit was 1/100,000.

## Discussion

The major finding in the present study was that the Zhaoyuan population, which experienced long-term nutritional deficiencies from childhood to adolescence, had increased risk for stomach cancer 15 to 20 years after the 1959–1961 Chinese famine. The birth cohorts who were exposed to famine or experienced malnutrition had higher stomach cancer mortality rates in later life than the birth cohorts not exposed to malnutrition. For the first time, we reported that exposure to famine in early life may increase the risk of gastric cancer in later life.

### Nutritional deficiency and stomach cancer risk

Zhaoyuan County is located 120° 08' to 120° 38' E and 37° 05' to 37° 33' N in the Shandong Peninsula. Before the era of economic reformation and open policy in China, the county was a poor agricultural location given its mountainous terrain with infertile soils. The annual average net income of farmers was less than USD50.

The trend in stomach cancer mortality in Zhaoyuan County is identical to that in the entire Shandong Province and China [6,46]. Over the past three decades, the survival for the disease has significantly improved. However, from 1970 to 1990, the five-year survival rates for patients with stomach cancer were 20% to 30% [47,48]. This finding may have an insignificant impact on the mortality trend from the long-term observation in the present study.

To support the above conclusion, information on long-term nutritional deficiency needs to be produced to fulfill the objective of the present study. From the establishment of the People’s Republic of China in 1949 until 1987, which entailed agricultural and societal reforms, the food of farmers was rationed by local rural community governments. This system of food assignment, which was unique to China, was recorded in the county annals.

World War II and a long civil war did not cause the Chinese people to suffer from nutritional deficiencies until the middle of the 1970s. The 1959–1961 Chinese famine was the most serious famine in the 20 th century. In 1960 and 1961, the aggregate per capita production of grain, which is the main component of the Chinese diet, fell by 51.39% and 40.97%, respectively, relative to that in 1959 (144 kg per capita) [49].

The severity of the 1959–1961 Chinese famine varied across regions and affected rural areas disproportionately [21,50]. Also, its duration varied geographically. The duration for most areas was from 1959 to 1961, but, for some of the worst areas it was from 1959 to 1963 or later [51]. The famine in Zhaoyuan County was one of the worst and its duration was from 1959 to 1963.

From 1955 to 1959, sweet potatoes accounted for 29.7% of the total food consumed by Zhaoyuan residents. In the winter of 1959, the sudden drop in temperature and misgovernance resulted in sweet potato damage, and consequently, severe food shortages. The government attempted to alleviate this situation by providing 100 g of grain per person per day. Inevitably, people still experienced malnutrition. The average caloric levels per capita from 1960 to 1961 were near the average survival level for Zhaoyuan residents (Figure 2).

Moreover, unlike any other famine in history, the 1959–1961 Chinese famine did not result in mass migration. The central and local governments prevented the migration of famine-stricken populations to other regions in search of food [33]. In the present study, the results of the APC analyses indicated that severe nutritional deficiency during early life may increase the risk for developing stomach cancer in later life. Therefore, a credible association between gastric cancer death rates and exposure to malnutrition indeed exists.

### Interpretable mechanisms

Subtle nutritional stimuli during specific critical developmental periods in early life, including the prenatal and early postnatal periods, can accelerate the carcinogenic response and induce persistent changes in gene expression as well as metabolism via the modulation of genetic and epigenetic mechanisms. However, this hypothesis needs further investigation.

Human risk factors for gastric cancer include *H. pylori*, dietary intake of those same N-nitrosamines or of nitrite which forms those N-nitrosamines in the stomach. Physiological actions of *H. pylori* colonization enhance the carcinogenic effect of N-nitrosamines delivered by smoking or dietary sources. This effect is modulated by host inflammatory response to the organism, by various virulence and other properties of the Helicobacter itself, and by host-organism interactions [52].

In 1989 we reported that less vitamin B1 in dietary intake and heavy alcohol drinking were associated with statistically significant increased risk for patients with atrophic gastritis at age 30–65 years [53]. At the same time, our colleagues found that the prevalence of persons infected by *H. pylori*[54] and the nitrite concentrations in a normal person’s gastric juice and drinking water were significantly higher in the high-incidence area than that in the low-incidence area of stomach cancer in Shandong Province [55].

Nutritional deficiency also predisposes to *H. pylori* infection. Many studies have demonstrated that this infection plays an important role in the development of noncardia gastric cancer [56,57]. A few studies in China have shown that this infection increases the risk of stomach cancer, and that the elimination of this infection from the stomach by drugs can decrease the risk [58-60].

A recent study has shown that CD74 expression in the gastric carcinoma cell line is related to *H. pylori* adhesion [6]. On the other hand, under a famine situation, people consume low-nutrition foods such as citrus coumarin, or risky foods such as salted meat. Consequently, the probability of *H. pylori* bacteria invading the epithelial cells of the stomach mucosa increases due to the high expression of CD74.

However, an EPIC cohort study has indicated that adherence to a Mediterranean diet is associated with a significant reduction in the risk of gastric adenocarcinoma (hazard ratio = 0.67; 95% CI = 0.47 and 0.94) [61]. On the other hand, a review of *H. pylori* infection in the Middle East shows that the prevalence of this infection in this region is similar to that in developing countries [62]. This finding implies that *H. pylori* invasion into the epithelial cells of the gastric mucosa may be a key factor for increased gastric cancer risk. The hypothesis is supported by recent study which found that malnutrition immediately after surgery may play a significant role in the development of wound complications [63].

Human risk factors for gastric cancer also include alcohol beverages [64]. Acetaldehyde derived from the alcoholic beverage itself and endogenously formed from ethanol has recently been classified by the International Agency for Research on Cancer/World Health Organization as a group 1 carcinogen to humans [65]. ALDH2 gene polymorphisms were found to modify the susceptibility to the development of gastric cancer associated with alcohol intake, especially in case of the ALDH2 *1/*2 genotype. The findings suggest an alcohol-ALDH2 genotype interaction in gastric carcinogenesis [66].

In summary, a possibly explanation for the association between exposure to famine and stomach cancer is that famine exposure in the early life damaged the mucous membrane of stomach or made it suffer tremendous loss in strength or resources so that some carcinogens including *H. pylori*, N-nitrosamines and acetaldehyde could easily intrude into the mucosa. Moreover, polymorphisms of some genes coordinated in the attack to develop the mucosa cells malignant mutation. The long-term carcinogenic process may consist of initial malnutrition stimulation and followed with reserve exposure to some risk factors for high-susceptibility persons of the disease.

## Conclusion

The results of the present study suggested that severe nutritional deficiency during early life may increase the stomach cancer risk. The birth cohorts who were exposed to famine or malnutrition had higher stomach cancer mortality rates in later life than the birth cohorts who were not exposed to malnutrition. This finding may guide the etiological research for the association between some nutritional components and stomach cancer development.

Limitation of this study is that the observation time is not long enough to evaluate age 50 years and over without famine exposure. Therefore, further follow up of the mortality and incidence of stomach cancer for those cohorts is needed to get the final evidence of the famine to be associated with the risk of stomach cancer development.

## Competing interests

There are not any conflicts of interest to declare for all authors of the manuscript.

## Authors’ contributions

Members listed below made their respective contributions to this manuscript. Professor QDL and HL designed the skeleton of this study, supervised the epidemiological survey, performed the statistical analysis and drafted the manuscript. JFL, MSW, JZL, JH, QHL, XJM and DNW carried out the data investigation, entered it and analysis. All authors read and approved the final manuscript.

## Pre-publication history

The pre-publication history for this paper can be accessed here:

http://www.biomedcentral.com/1471-2407/12/315/prepub
